# Supplementary material for: CMR reveals myocardial damage from cardiotoxic oncologic therapies in breast cancer patients
Source: Int J Cardiovasc Imaging. 2023 Nov 25;40(2):225–35. doi: 10.1007/s10554-023-02996-7 (PMC10884136; doi:10.1007/s10554-023-02996-7)
Supplement: Supplementary file 1 — Supplementary Material 1 [file 10554_2023_2996_MOESM1_ESM.docx]

Table S1. Logistic regression analysis to predict mild cancer therapy related cardiac dysfunction.

| **Value** | **OR (95% CI)** | **β coefficient value** | **p-value** |
| --- | --- | --- | --- |
| Age | 0.99 (0.92, 1.06) | -0.01 | 0.739 |
| Body mass index | 1.19 (0.99, 1.43) | 0.17 | 0.066 |
| Hypertension | 1.05 (0.19, 5.69) | 0.05 | 0.967 |
| Dyslipidemia | 1.63 (0.13, 20.00) | 0.49 | 0.705 |
| Current or Ex-smoking | N/A | N/A | 0.999 |
| Previous cancer therapy | 2.60 (0.24, 28.15) | 0.96 | 0.432 |
| Medication |  |  |  |
| Betablockers | 2.60 (0.24, 28.15) | 0.96 | 0.432 |
| ACE inhibitors/AT1 receptor blockers | 3.71 (0.37, 37.71) | 1.31 | 0.267 |
| Statins | 1.63 (0.13, 20.00) | 0.49 | 0.705 |
| Tumour biology |  |  |  |
| T-Stadium > T1 | 0.55 (0.11, 2.73) | -0.61 | 0.460 |
| N-Stadium > N0 | 0.87 (0.21, 3.71) | -0.14 | 0.854 |
| Ki67 expression | 1.00 (0.98, 1.03) | 0.00 | 0.903 |
| Her2 positivity | 0.71 (0.14, 3.56) | -0.34 | 0.681 |
| ER positivity | 2.83 (0.67, 12.02) | 1.04 | 0.159 |
| PR positivity | 1.18 (0.29, 4.88) | 0.16 | 0.821 |
| Anti-tumour therapy |  |  |  |
| Anthracyclines | 2.83 (0.23, 34.92) | 1.04 | 0.416 |
| Her2 targeted therapy | 0.71 (0.14, 3.56) | -0.34 | 0.681 |
| Anthracycline combined with Her2 targeted therapy | 0.73 (0.12, 4.35) | -0.31 | 0.733 |
| Radiotherapy | 0.43 (0.07, 2.67) | -0.84 | 0.367 |
